# Cross-talk between aging resilience pathways and autoimmunity onset

**DOI:** 10.3389/fimmu.2025.1712575

**Published:** 2025-12-09

**Authors:** Rahul Mittal, Danay Saavedra, Mannat Mittal, Khemraj Hirani

**Affiliations:** 1 Diabetes Research Institute, University of Miami Miller School of Medicine, Miami, FL, United States; 2 Division of Endocrinology, Diabetes, and Metabolism, Department of Medicine, University of Miami Miller School of Medicine, Miami, FL, United States

**Keywords:** aging, biological resilience, autoimmunity, inflammaging, senescence, healthspan, longevity

## Abstract

Aging and autoimmunity intersect through the progressive decline of resilience pathways that maintain physiological stability. Resilience refers to the integrated capacity of molecular, cellular, and systemic mechanisms to repair damage, adapt to stress, and preserve immune tolerance. With advancing age, resilience deteriorates due to chronic inflammation, cellular senescence, epigenetic drift, and metabolic dysfunction. While a wide spectrum of autoimmune diseases exists, this perspective focuses primarily on those that emerge or progress with advancing age, in which a decline in immune resilience rather than congenital immune defects plays the predominant role. These changes weaken adaptive capacity and promote conditions that allow autoreactive lymphocytes to persist, initiating autoimmune pathology. This perspective frames autoimmunity as a sentinel manifestation of resilience collapse rather than an isolated failure of immune tolerance. The objective of this article is to delineate the shared molecular and systemic mechanisms by which age-associated loss of resilience promotes autoimmune susceptibility, and to highlight how this framework can guide both research priorities and therapeutic innovation. By examining convergent pathways across inflammation, senescence, epigenetics, and metabolism, we emphasize that autoimmune disease arises from integrated failures in the networks that sustain homeostasis. Recognizing these connections enables the development of integrated biomarkers to detect resilience decline and identify individuals at risk before clinical onset. It further supports therapeutic strategies aimed at enhancing repair capacity, maintaining immune tolerance, and restoring adaptive responses. Recasting autoimmunity in this framework provides opportunities for preventive interventions and novel treatments with the potential to extend healthspan.

## 1 Introduction

Biological resilience in the context of aging refers to the dynamic ability of an organism to sustain physiological stability, restore equilibrium after perturbation, and adapt to a wide variety of environmental and internal stressors throughout life (1–5). This concept extends beyond the mere absence of disease and captures the integrated function of molecular, cellular, and systemic mechanisms that preserve healthspan and support longevity (6–10). Resilience encompasses processes such as efficient DNA repair, maintenance of proteostasis, control of oxidative stress, balanced inflammatory signaling, preservation of metabolic flexibility, and the fine regulation of immune responses (11–14). Together, these mechanisms act as adaptive buffers that prevent transient challenges from progressing into irreversible tissue damage or chronic pathological states. With advancing age, a progressive decline in these resilience pathways reduces the capacity to repair molecular insults, compromises immune tolerance, and heightens susceptibility to chronic inflammatory and degenerative conditions (15–19). This chronic, low-grade inflammatory state has been termed inflammaging, reflecting the persistent activation of innate immune pathways and dysregulated cytokine networks that characterize the aging process and contribute to multiple age-related pathologies (20–24).

Autoimmunity provides a striking example of the consequences of resilience dysfunction. The immune system is inherently designed to discriminate between self and non-self, relying on multiple tolerance mechanisms to prevent autoreactive lymphocytes from initiating pathogenic responses (25). Central tolerance, established during lymphocyte development, and peripheral tolerance, maintained through regulatory T cells, anergy, and immune checkpoints, together ensure that immune surveillance does not turn against host tissues (26). When these tolerance mechanisms falter, autoreactive T and B cells escape regulation, leading to the chronic inflammation and tissue destruction characteristic of autoimmune diseases (27–29). Importantly, these failures rarely occur in isolation. They are frequently precipitated or exacerbated by broader age-associated deficits in resilience pathways, including persistent low-grade inflammation, accumulation of senescent immune and stromal cells, mitochondrial dysfunction, and progressive instability of the epigenetic landscape (30–34).

Although autoimmunity is often described as increasing with chronological age, population-level analyses reveal a bimodal distribution across the lifespan (35). Diseases such as type 1 diabetes, multiple sclerosis, inflammatory bowel disease, and lupus predominate during youth and early adulthood, whereas conditions including rheumatoid arthritis, Sjögren’s syndrome, vasculitis, and autoimmune thyroid disease become more prevalent in later decades. This bimodality indicates that distinct resilience mechanisms may govern immune tolerance at different life stages, with early life developmental resilience differing from late life cumulative resilience erosion. Accordingly, defining the temporal windows of resilience decline is essential to contextualize when and how aging-related processes contribute to autoimmune susceptibility.

In this perspective, “autoimmunity” refers specifically to immune-mediated diseases in which the loss of self-tolerance arises gradually through cumulative molecular and systemic stress, rather than from congenital or monogenic defects of immune regulation. Classic examples include rheumatoid arthritis, multiple sclerosis, systemic lupus erythematosus, and autoimmune thyroiditis, which often exhibit age-associated onset or exacerbation. This definition deliberately distinguishes resilience-linked autoimmunity from early-onset conditions such as type 1 diabetes in childhood, in which inborn defects in thymic selection or immune checkpoint function play dominant causal roles (36). Our goal is therefore to focus on the subset of autoimmune phenomena that emerge from age-related erosion of adaptive capacity and homeostatic resilience.

**Figure 1:**
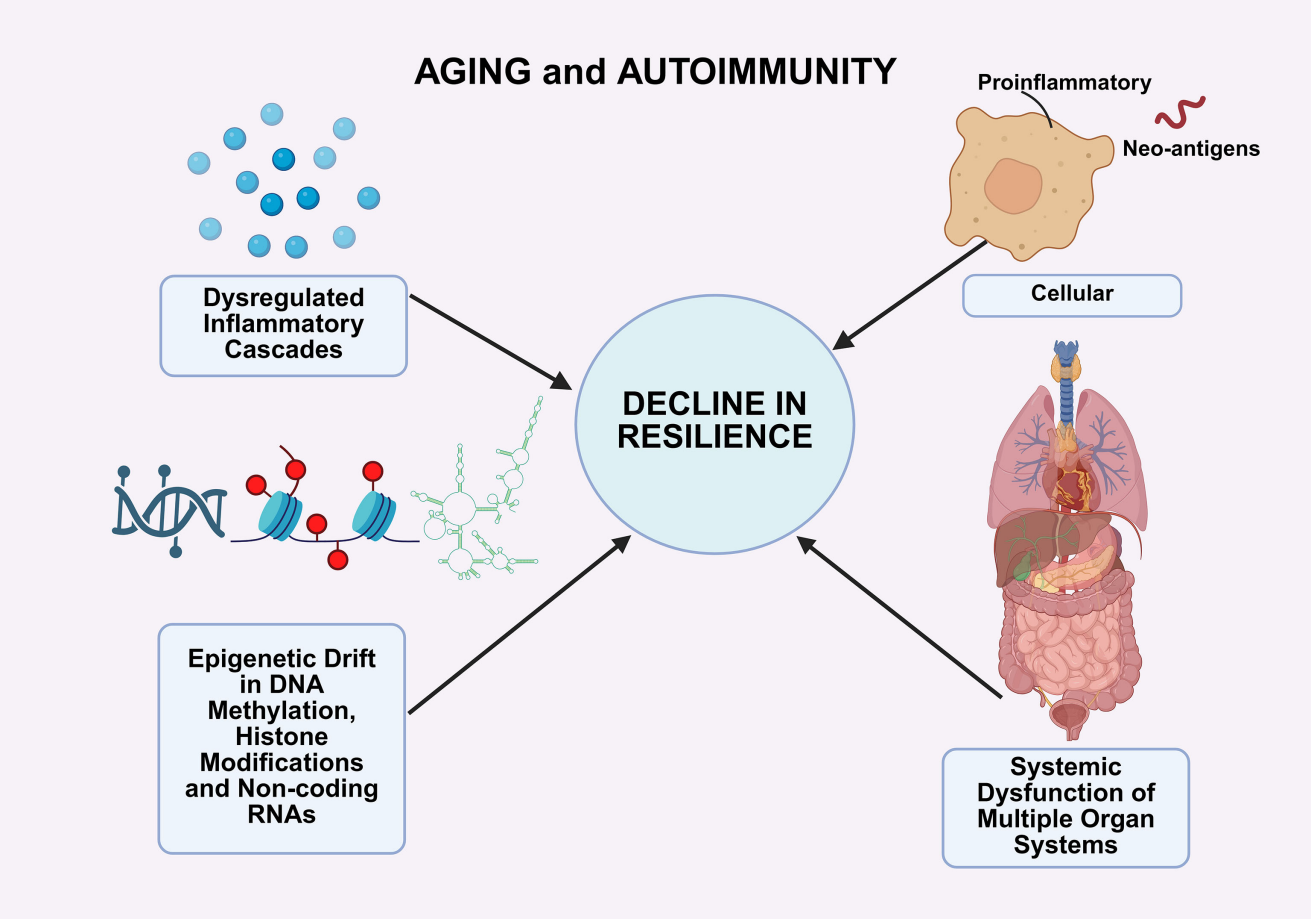
Aging-associated decline in resilience promotes autoimmune susceptibility.Advancing age leads to progressive impairment of resilience pathways that sustain physiological stability and immune tolerance. Chronic inflammatory cascades establish a persistent low-grade inflammatory milieu that exhausts regulatory networks and facilitates autoreactive lymphocyte persistence. Accumulation of senescent immune and stromal cells generates a secretory phenotype enriched in pro-inflammatory mediators and neoantigen exposure, further amplifying immune activation. Epigenetic drift in DNA methylation, histone modifications, and non-coding RNA expression destabilizes transcriptional programs required for quiescence and tolerance. At the systemic level, declining resilience diminishes the capacity of organ systems to buffer immune dysregulation, thereby enabling the transition from transient autoreactivity to overt autoimmune disease. Created in BioRender. Mittal, R. (2026) https://BioRender.com/hdef6ty.

The central theme of this perspective is that the biology of aging and the pathogenesis of autoimmunity are not separate domains but instead represent interconnected manifestations of resilience decline (**Figure 1**). At the molecular level, dysregulated inflammatory cascades that contribute to the phenomenon of inflammaging also drive the loss of self-tolerance (37, 38). At the cellular level, senescent cells secrete pro-inflammatory mediators and expose neo-antigens that perpetuate autoimmune activity (39). At the epigenetic level, drift in DNA methylation patterns, histone modifications, and non-coding RNA networks destabilizes transcriptional programs that normally enforce immune quiescence (40, 41). At the systemic level, the dysfunction of resilience undermines the ability of multiple organ systems to compensate for immune dysregulation, thereby facilitating the onset of clinically overt autoimmune disease.

To strengthen the conceptual framework presented here, we propose a more operational definition of immune resilience. Immune resilience refers to the ability of the immune system to recover its functional stability after perturbation, and it can be quantified through a set of multidimensional biological measurements (42, 43). Indicators of repair capacity, including γH2AX resolution kinetics, maintenance of proteostatic integrity, mitochondrial performance, NAD^+^ availability, and respiration profiles, provide insight into how effectively cells restore homeostasis after molecular injury (44, 45). The overall organization of the immune compartment, reflected in the proportions of effector, regulatory, and senescent populations, further indicates whether immunological balance is preserved (23, 46). Parameters such as the frequency of regulatory T cells, the accumulation of senescence-associated T and B cells, and the magnitude of the senescence-associated secretory phenotype are particularly informative in this regard (47). Functional assays that monitor recovery after stimulation, including the return of cytokine trajectories to baseline concentrations and the restoration of transcriptional equilibrium following ex vivo challenge, offer an additional dimension for assessing the system’s ability to reset after stress (48). Integrated biomarker profiles that include circulating inflammatory mediators such as IL-6 and C-reactive protein, epigenetic age acceleration indices, T- and B-cell repertoire diversity, and metabolic or proteomic signatures associated with adaptive capacity provide a systems-level perspective on resilience (49). Together, these measurable features create a coherent framework for evaluating immune resilience over time, enabling comparison across individuals and age groups and allowing these metrics to be linked with the initiation or progression of autoimmune disease. Incorporating such operational measurements renders the concept of resilience both biologically well-defined and clinically actionable, supporting its use in risk stratification, early disease detection, and therapeutic monitoring.

In the context of resilience-directed therapeutic strategies, the term restoring adaptive responses refers to two biologically distinct processes. The first concerns improving the functional competence of the existing adaptive immune-cell pool, including enhanced effector function, regulatory capacity, metabolic adaptability, and the ability to return to immunological equilibrium following stimulation. The second concerns reestablishing the ability of the immune system to generate a diverse repertoire of new lymphocytes through mechanisms such as thymic activity, maintenance of naïve T and B cell populations, and preservation of receptor diversity. Distinguishing these processes is important, because aging alters functional responsiveness and generative capacity through partially independent pathways, and interventions designed to enhance resilience may differentially affect them.

Reframing autoimmunity as a manifestation of resilience collapse opens a novel conceptual framework for understanding the biology of immune aging. Rather than viewing autoimmune diseases solely as disorders of aberrant immune activation, they can be interpreted as sentinel conditions that reveal broader vulnerabilities in the resilience architecture of the aging organism. This perspective has profound implications for both mechanistic research and therapeutic innovation. By investigating the cross-talk between resilience pathways and autoimmune susceptibility, it may be possible to identify biomarkers that predict loss of tolerance before disease onset, to design interventions that restore resilience rather than only suppressing inflammation, and ultimately to develop strategies that extend healthspan by preserving the adaptive capacity of the immune system.

## 2 Shared molecular and cellular pathways

The intersection between aging-associated resilience pathways and the onset of autoimmunity can be most effectively comprehended by examining the molecular and cellular processes that underpin both phenomena. Four key domains, inflammation, cellular senescence, epigenetic regulation, and metabolic function, recurrently emerge as central determinants of whether biological systems maintain homeostasis with age or succumb to autoimmune dysregulation (**Table 1**).

**Table 1:** Molecular and cellular pathways at the intersection of aging resilience and autoimmunity.

Domain	Role in resilience	Contribution to autoimmunity	Examples/key mediators
Inflammation (Inflammaging)	Resolves acute damage, supports host defense	Chronic IL-6, TNF-α, IL-1β promote autoreactive T cells	NLRP3 inflammasome, CRP
Cellular Senescence	Limits proliferation of damaged cells	SASP fuels inflammation, presents neo-antigens	IL-6, IL-8, MMPs
Epigenetic Regulation	Maintains transcriptional stability and immune tolerance	Drift enables aberrant inflammatory gene expression	DNA hypomethylation, miR-21, loss of GAS5
Metabolic Pathways	Energy balance, stress recovery, autophagy	Dysfunction biases immune cells toward pro-inflammatory phenotypes	NAD^+^ depletion, mitochondrial dysfunction, defective autophagy

IL, Interleukin; TNF-α, Tumor necrosis factor-alpha; IL-1β, Interleukin-1 beta; NLRP3, NOD-, LRR- and pyrin domain-containing protein 3; CRP, C-reactive protein; SASP, Senescence-associated secretory phenotype; MMPs, Matrix metalloproteinases; miR, microRNA; GAS5, Growth arrest-specific 5; NAD^+^, Nicotinamide adenine dinucleotide.

### 2.1 Inflammation and inflammaging

One of the most extensively characterized hallmarks of aging is the gradual establishment of a chronic, low-grade inflammatory state often referred to as inflammaging (50). Unlike the acute, tightly regulated inflammatory responses that are essential for pathogen clearance and tissue repair, inflammaging is characterized by persistent elevations of pro-inflammatory mediators such as interleukin-6, tumor necrosis factor-alpha, and C-reactive protein (51, 52). This chronic inflammatory environment undermines resilience by exhausting immune regulatory networks, disrupting tissue repair capacity, and amplifying oxidative stress. In the context of autoimmunity, the same inflammatory mediators that impair resilience also serve as potent drivers of autoreactivity. The NLRP3 inflammasome, for example, represents a critical node at which resilience and autoimmunity converge (53). The NLRP3 (NOD-, LRR-, and pyrin domain-containing protein 3) inflammasome is a multiprotein sensor complex expressed predominantly in myeloid-lineage cells such as monocytes, macrophages, and dendritic cells, with lower but functionally relevant expression also found in endothelial, epithelial, and certain lymphoid cells (54–57). As a cytosolic pattern-recognition receptor, NLRP3 detects diverse pathogen- and damage-associated molecular patterns, including extracellular ATP, crystalline particulates, and mitochondrial stress signals (58, 59). Upon activation, NLRP3 nucleates the inflammasome complex with ASC and caspase-1, leading to the maturation and secretion of interleukin-1β and interleukin-18 and, in some contexts, to pyroptotic cell death (60–62). Through these mechanisms, NLRP3 functions as a central hub linking innate immune sensing, sterile inflammation, and tissue homeostasis (50, 51, 63–65). Persistent or dysregulated activation of this inflammasome with age results in excessive interleukin-1β and interleukin-18 production, accelerating tissue degeneration and promoting differentiation of autoreactive T-helper subsets (66–68). Thus, NLRP3 exemplifies the point at which resilience pathways and autoimmune activation converge within the broader landscape of inflammaging.

While activation of NLRP3 is necessary for host defense and tissue recovery, persistent activation with age results in excessive production of interleukin-1β and interleukin-18, which not only accelerate tissue degeneration but also promote differentiation of autoreactive T helper cell subsets (63, 69). Thus, inflammaging exemplifies a double-edged process in which the very molecules designed to sustain resilience under stress become catalysts for autoimmune activation when regulation fails.

### 2.2 Cellular senescence

Another prominent feature of aging is the accumulation of senescent cells within both immune and stromal compartments (70–73). Senescence serves as an initially protective mechanism that prevents uncontrolled proliferation of damaged or stressed cells (74). However, when senescent cells persist, they adopt a senescence-associated secretory phenotype characterized by the release of pro-inflammatory cytokines, chemokines, growth factors, and matrix-degrading enzymes (75). For example, senescent fibroblasts in synovial tissue of patients with rheumatoid arthritis secrete interleukin-6 (IL-6), interleukin-8 (IL-8), and matrix metalloproteinases (MMPs), sustaining inflammation, recruiting immune cells, and contributing to joint destruction (34, 76) This secretory profile undermines resilience by impairing tissue architecture, promoting chronic inflammation, and disrupting regenerative niches. At the same time, the senescence-associated secretory phenotype facilitates autoimmune pathology by increasing the presentation of neo-antigens, enhancing autoreactive lymphocyte recruitment, and diminishing the clearance of self-reactive clones (76). Moreover, the age-associated decline in immune surveillance impairs the removal of senescent cells, further amplifying this cycle (77). The resulting environment simultaneously weakens the organism’s capacity for adaptation and repair while priming the immune system for self-directed attack.

To fully understand how senescence influences resilience and autoimmune activation, it is essential to consider not only stromal cell senescence but also the senescence of immune populations (78, 79). Immune cells exhibit characteristic age-associated alterations that parallel those seen in stromal tissues, including impaired proliferative capacity, reduced receptor repertoire diversity, disrupted metabolic adaptability, and increased secretion of pro-inflammatory mediators (78, 80, 81). These senescent immune populations frequently develop a secretory profile analogous SASP, thereby reinforcing chronic inflammatory signaling and weakening the regulatory mechanisms that normally sustain immune tolerance (82, 83). In addition, senescent T cells, B cells, natural killer cells, and myeloid subsets demonstrate diminished ability to re-establish functional homeostasis following antigenic or inflammatory stimulation, which reflects a measurable decline in immune-system resilience (81, 84). This reduced capacity for recovery permits autoreactive lymphocytes that would ordinarily be suppressed or eliminated to persist within the immune repertoire (85). Incorporating immunosenescence into the conceptual framework of resilience therefore emphasizes that age-related functional decline occurs concurrently across both stromal and immune compartments, and that the interaction of these processes plays a central role in determining susceptibility to autoimmune activation.

### 2.3 Epigenetic regulation

Epigenetic integrity is a cornerstone of biological resilience, ensuring that transcriptional programs remain stable in the face of environmental and metabolic stressors (86, 87). With aging, however, there is progressive epigenetic dysregulation in DNA methylation, histone modifications, and non-coding RNA networks (88–93). These changes destabilize resilience by eroding transcriptional fidelity, compromising lineage identity, and reducing the adaptability of immune and stromal cells (94–96). The same epigenetic dysregulation also precipitates autoimmunity (33, 97). For example, hypomethylation at promoters of pro-inflammatory genes results in their aberrant expression in autoreactive T cells (98–100). MicroRNAs such as miR-21 are frequently upregulated in aging and autoimmune contexts, where they suppress DNA methyltransferase activity, leading to global DNA hypomethylation and derepression of inflammatory genes (101–103). Conversely, protective non-coding RNAs such as the long non-coding RNA GAS5 are often depleted, which removes critical checkpoints on T cell activation and immune tolerance (104–106). These examples highlight how loss of epigenetic resilience not only destabilizes transcriptional regulation but directly enables autoimmune phenotypes (107–109).

### 2.4 Metabolic pathways

Metabolic homeostasis is increasingly recognized as a fundamental determinant of resilience in aging (110). Efficient energy production, redox balance, and nutrient sensing allow cells to withstand stress and recover from injury (111, 112). With age, mitochondrial dysfunction, accumulation of oxidative damage, and depletion of nicotinamide adenine dinucleotide undermine these processes, thereby weakening resilience (113–115). Impaired autophagy further exacerbates this decline by allowing the buildup of damaged proteins and organelles that perpetuate cellular stress (116–119). In immune cells, these metabolic defects have direct consequences for autoimmunity (120, 121). Mitochondrial dysfunction skews T cell differentiation toward pro-inflammatory phenotypes, while reduced nicotinamide adenine dinucleotide availability impairs the activity of sirtuins that normally suppress inflammatory transcriptional programs (122). At the same time, defective autophagy allows persistence of damaged self-antigens that can serve as triggers for autoreactive immune responses (123, 124). For example, in type 1 diabetes, impaired autophagy in pancreatic β cells leads to accumulation of damaged mitochondria and misfolded proteins that enhance β-cell immunogenicity, promoting T cell–mediated autoimmune destruction of islets (125, 126). Collectively, these metabolic failures diminish the ability of the organism to adapt to stress while simultaneously sustaining the survival and expansion of autoreactive lymphocytes.

Taken together, these overlapping pathways illustrate that the molecular mechanisms governing resilience in aging and those driving autoimmune susceptibility are not independent but rather deeply interconnected. Inflammation, senescence, epigenetic drift, and metabolic dysfunction each undermine the capacity to maintain equilibrium, and in doing so, they open the door to autoreactive immune responses. Recognizing this convergence provides a critical framework for rethinking both the biology of aging and the origins of autoimmune disease.

### 2.5 B cell function and autoantibody production

B cells play a central role in the pathogenesis of many autoimmune diseases through the production of autoantibodies, antigen presentation, and cytokine secretion (127–130). The generation of autoreactive B cell clones reflects both defective central tolerance in the bone marrow and impaired peripheral checkpoints that normally eliminate or silence self-reactive cells (131, 132). With advancing age, several resilience-associated processes such as altered germinal center dynamics, reduced regulatory B cell function, and chronic inflammatory signaling can disrupt B cell homeostasis and favor autoantibody production (133–135). However, while B cell dysregulation is a hallmark of autoimmune pathology, its contribution to resilience decline may be largely secondary to upstream failures in immune regulation, metabolic balance, and inflammatory control that create permissive conditions for autoreactive clones to persist. Consequently, in this perspective, B cell involvement is acknowledged as a downstream manifestation of resilience erosion rather than a primary driver of its decline. If certain autoimmune conditions are predominantly mediated by autoantibodies, such as lupus or myasthenia gravis, these may represent disease subsets in which resilience collapse amplifies rather than initiates humoral autoreactivity.

### 2.6 T cell biology and immune resilience

T cells constitute the central regulatory axis of adaptive immunity and are critical determinants of immune tolerance and resilience. Thymic selection, peripheral regulatory circuitry, and the metabolic and epigenetic programs that sustain T cell homeostasis collectively establish the capacity of the system to prevent autoreactivity (136). Aging perturbs each of these domains. Progressive thymic involution contracts the naïve T cell pool and reduces TCR repertoire diversity, limiting the organism’s ability to replace dysfunctional or autoreactive clones (137). Concurrently, age-associated epigenetic drift destabilizes transcriptional networks that maintain quiescence and lineage fidelity, predisposing CD4^+^ and CD8^+^ T cells to aberrant activation (138). Mitochondrial dysfunction and impaired redox balance further bias differentiation toward pro-inflammatory Th1 and Th17 phenotypes while compromising the stability and suppressive capacity of regulatory T cells (139–141). In parallel, the accumulation of senescent and terminally differentiated T cell subsets, marked by reduced proliferative potential and a pro-inflammatory secretory profile, diminishes the ability of system to resolve immune perturbations and restore equilibrium after stress (47).

Collectively, these alterations represent a progressive erosion of T cell–intrinsic resilience mechanisms that ordinarily buffer against transient antigenic or inflammatory insults. Their decline increases the persistence, survival, and effector potential of autoreactive T cells, thereby lowering the threshold for autoimmune activation. Integrating these processes into our framework reinforces the central thesis that late-onset autoimmunity reflects not an isolated failure of tolerance but the cumulative breakdown of resilience pathways that regulate T cell generation, maintenance, and functional recalibration across the lifespan.

## 3 Systems-level interactions

The resilience framework may accommodate the possibility that distinct phases of resilience erosion contribute to autoimmunity. For example, resilience mechanisms tied to thymic education, metabolic plasticity, and epigenetic programming may decline as early as the second decade of life, favoring early-onset diseases such as type 1 diabetes, whereas cumulative inflammatory and senescent burden emerging after midlife may underlie later-onset disorders like rheumatoid arthritis and Sjögren’s syndrome. While molecular and cellular mechanisms provide the foundation for understanding how resilience pathways influence the onset of autoimmunity, it is at the systems level that the cumulative impact of these processes becomes clinically apparent. The organism can be viewed as a network of interconnected subsystems in which resilience operates as a buffer, delaying or preventing the transition from physiological adaptation to pathological breakdown. When these buffering capacities are compromised, the tipping point into autoimmunity is reached more readily, and localized perturbations may escalate into systemic disease.

### 3.1 Resilience as a buffer against autoimmunity

Biological resilience serves as an integrative defense mechanism that protects the organism from the consequences of immune dysregulation (142–144). In youth and early adulthood, robust resilience pathways compensate for molecular insults, limit the consequences of inflammatory bursts, and rapidly restore immune tolerance after perturbations (145, 146). For example, transient autoreactive responses that arise after infection are typically neutralized by the coordinated actions of regulatory T cells, tolerogenic dendritic cells, and efficient DNA repair and epigenetic stability (147–150). With aging, however, these buffering systems decline (146, 151). The reduced capacity to resolve inflammation, the accumulation of senescent immune and stromal cells, and the diminished ability to reset epigenetic programs after stress all weaken this protective barrier (145, 152, 153). As a result, autoreactive immune cells that would normally be suppressed or eliminated may persist, gradually eroding immune tolerance and predisposing the individual to autoimmune pathology (109, 154–156).

### 3.2 Cross-talk loops between pathways

The decline of resilience does not occur in isolated domains but rather through a web of reinforcing feedback loops that accelerate the transition from adaptation to pathology (157–159). Chronic low-grade inflammation, characteristic of inflammaging, is a prime example (20, 160–162). Persistent inflammatory signaling not only erodes resilience by damaging tissues and exhausting regulatory immune pathways, but it also drives epigenetic drift (163–165). Aberrant DNA methylation and histone modifications induced by inflammatory cytokines destabilize transcriptional programs that normally maintain immune quiescence (166–168). This in turn promotes clonal expansion of autoreactive T and B cells, which perpetuate further inflammation, thereby completing a vicious cycle (169–171). Metabolic dysfunction integrates into this loop by reducing mitochondrial fitness and impairing autophagy, thereby increasing the load of damaged self-antigens that amplify autoreactive responses (125, 126, 172). Each of these processes feeds forward into the others, creating a system in which resilience pathways are progressively dismantled and the probability of autoimmune onset is markedly increased (33, 169, 173).

### 3.3 Organ and tissue specificity

Recent evidence highlights that resilience erosion and autoimmune activation manifest through tissue-specific vulnerabilities rather than uniform systemic decline. For example, in the pancreas, impaired β-cell autophagy and limited regenerative capacity heighten susceptibility to T-cell–mediated destruction, as seen in type 1 diabetes (174, 175). Certain organs appear more vulnerable because their intrinsic resilience reserves are relatively limited or because they are disproportionately affected by age-associated stressors (176). The pancreas is a prime example, as the autoimmune destruction of insulin-producing beta cells in type 1 diabetes highlights the inability of this tissue to regenerate once tolerance mechanisms are breached. In the central nervous system, age-related microglial senescence and mitochondrial dysfunction reduce metabolic flexibility and promote chronic neuroinflammation, contributing to autoimmune demyelination in diseases such as multiple sclerosis (177). Joints and connective tissues illustrate another paradigm, where persistent biomechanical stress synergizes with senescent fibroblast accumulation and inflammatory signaling to sustain rheumatoid arthritis pathology (178, 179). Multi-organ aging studies reveal that the rate and pattern of resilience loss differ across tissues, with metabolic and immune organs showing the earliest and most pronounced signatures of decline.

At the systems level, these localized changes are interconnected through inflammatory, metabolic, and epigenetic feedback loops that propagate stress signals across organ systems (180–184). Declining mitochondrial function in immune cells, for instance, amplifies systemic cytokine production, which in turn accelerates senescence and impairs repair mechanisms in distal tissues (180, 185, 186). This bidirectional communication transforms resilience loss in one tissue into a whole-organism vulnerability, establishing a self-reinforcing cycle that culminates in widespread immune dysregulation and autoimmune pathology.

## 4 Future directions and hypotheses

The recognition that resilience pathways and autoimmune susceptibility are deeply interconnected opens a wide range of opportunities for future research and clinical translation. Several promising directions can be identified with potential to transform both mechanistic understanding and therapeutic approaches to immune-mediated disease in aging.

### 4.1 Biomarkers of cross-talk between resilience and autoimmunity

A pressing need is the development of reliable biomarkers that capture the shared biological space between resilience decline and autoimmune onset (**Supplementary Table 1**). Current tools such as epigenetic clocks (187–189), which measure age-associated DNA methylation drift, provide insights into biological age but rarely incorporate immune-specific information. Future efforts should focus on composite biomarker signatures that integrate multiple dimensions of resilience biology. Such signatures may include non-coding RNA profiles that regulate both epigenetic stability and immune tolerance, quantitative measures of senescence-associated secretory factors that reflect the accumulation of dysfunctional cells, and longitudinal tracking of inflammatory mediators that signal erosion of adaptive capacity. A multidimensional biomarker framework would not only allow prediction of autoimmune risk but could also serve as a metric for evaluating the effectiveness of resilience-enhancing interventions. These integrated biomarkers may help identify individuals at high risk earlier and enable more personalized therapeutic strategies.

### 4.2 Therapeutic reframing through resilience enhancement

Current therapies for autoimmune diseases have historically relied on broad immunosuppressive agents that attenuate global immune activation. In recent years, however, targeted approaches including biologic and small-molecule disease-modifying antirheumatic drugs (DMARDs) have transformed clinical management by selectively blocking key cytokines, receptors, or intracellular signaling pathways (for example, anti–TNF, IL-6R, and JAK inhibitors) (190–194). While these advances limit the off-target effects of traditional immunosuppression, they remain primarily directed at dampening immune activation rather than restoring the underlying resilience pathways that sustain tolerance and repair. A future therapeutic paradigm should therefore aim to complement these targeted agents with interventions that enhance resilience itself (**Supplementary Table 2**). Potential strategies include the use of senolytic drugs to selectively eliminate senescent cells and reduce their pro-inflammatory secretory output. Examples of senolytic agents include the BCL-2 family inhibitor navitoclax (ABT-263), the combination of dasatinib plus quercetin that targets senescent endothelial and adipocyte progenitor cells, and the flavonoid fisetin, which promotes apoptosis of senescent immune and stromal cells (195–203). Additional classes under investigation include HSP90 inhibitors (such as ganetespib) and FOXO4–p53 interaction disruptors (such as FOXO4-DRI peptides), both of which restore tissue repair capacity and reduce the senescence-associated secretory phenotype in preclinical models (204–206).

Supplementation or pharmacological activation of nicotinamide adenine dinucleotide can further restore metabolic and mitochondrial function, while epigenetic modulators stabilize DNA methylation and histone landscapes in autoreactive immune cells. Nicotinamide adenine dinucleotide (NAD^+^) is a central coenzyme in redox reactions and a key regulator of cellular metabolism, mitochondrial function, and DNA repair (44, 207, 208). Age related declines in NAD^+^ levels contribute to impaired sirtuin activity, mitochondrial dysfunction, and increased inflammatory signaling, which are hallmarks of resilience erosion (209–212). NAD targeted therapies aim to replenish intracellular NAD^+^ pools or enhance their biosynthetic pathways, thereby restoring metabolic resilience (213–215). Strategies under investigation include supplementation with NAD^+^ precursors such as nicotinamide riboside (NR) and nicotinamide mononucleotide (NMN), inhibition of NAD^+^ consuming enzymes such as CD38, and activation of sirtuins that rely on NAD^+^ as a substrate (216). Preclinical studies demonstrate that these interventions can reduce inflammaging, improve mitochondrial biogenesis, and reestablish immune tolerance, highlighting their potential as resilience enhancing approaches in age associated autoimmunity (212, 217–221).

Emerging RNA-based therapies that target microRNAs or long non-coding RNAs may also provide unprecedented precision in re-establishing tolerance while sparing global immune suppression. For example, anti-miR-21 and anti–miR-155 oligonucleotides have demonstrated immunoregulatory effects in preclinical autoimmune models by dampening pathogenic Th17 and B-cell activation, while GAS5-mimetic lncRNA constructs are being developed to restore regulatory T-cell homeostasis and immune tolerance (105, 222–224). By directly supporting resilience pathways, these interventions may help delay autoimmune onset, reduce disease severity, and potentially extend healthspan.

### 4.3 Translational opportunities and advanced research methodologies

To translate these concepts into clinical impact, new approaches to human research are required. Longitudinal cohort studies that track individuals from early adulthood through late life, with systematic measurement of resilience markers alongside autoimmune activity, will be essential to map causal trajectories. Single-cell multiomics, integrating transcriptomic, epigenomic, proteomic, and metabolic data at the level of individual immune cells (225–228), will provide unprecedented resolution of the cross-talk between resilience pathways and autoreactive potential. Spatial multiomics could further reveal how resilience erosion interacts with local tissue environments, clarifying why certain organs such as the pancreas, central nervous system, and joints exhibit particular vulnerability. These approaches will also facilitate the identification of therapeutic targets and the refinement of personalized interventions.

### 4.4 Broader hypothesis: autoimmunity as a sentinel of resilience collapse

Autoimmunity can emerge through heterogeneous mechanisms across the human lifespan. However, the conceptual framework advanced here focuses primarily on late-onset forms that are mechanistically linked to the progressive erosion of biological resilience. These conditions are interpreted as emergent phenomena arising from cumulative failures across molecular, cellular, and systemic adaptive networks rather than from discrete congenital or developmental defects in immune regulation. Delineating this scope refines the mechanistic precision of our model and situates autoimmunity within the broader continuum of age-associated physiological decline. Within this defined context, autoimmune disease may be viewed as an early sentinel of resilience collapse, reflecting the heightened sensitivity of the immune system to perturbations in homeostatic control. As resilience pathways that normally sustain repair, tolerance, and adaptive recalibration deteriorate, immune dysregulation manifests as one of the earliest measurable indicators of systemic instability. In this view, autoimmunity represents not merely a discrete breakdown of immune tolerance but a systems-level indicator of diminishing resilience capacity. As the immune system is uniquely positioned at the intersection of molecular surveillance, cellular adaptation, and systemic integration, autoimmune activation functions as an early barometer of resilience failure. Molecular surveillance refers to the continuous monitoring of molecular integrity within cells and tissues, including the detection and repair of DNA damage, removal of misfolded or oxidized proteins, sensing of metabolic stress, and recognition of pathogen- or damage-associated molecular patterns by innate immune receptors. Aberrant self-recognition thus represents less a discrete immunological accident than a measurable tipping point in the broader capacity of the organism to withstand stress, repair injury, and recalibrate after perturbation.

This reframing has profound implications. First, it positions autoimmunity as a leading indicator of healthspan erosion, much like arrhythmias can signal cardiac instability before heart failure. Second, it highlights the value of autoimmune phenotypes as a research window into resilience dynamics, providing a natural model through which to dissect the interplay of stress response, repair capacity, and adaptive limits. Finally, it redirects therapeutic goals: rather than merely quelling inflammatory damage, interventions should be evaluated for their capacity to rebuild resilience architecture. This entails restoring feedback control, reinforcing tolerance checkpoints, and re-establishing systemic adaptability.

In this perspective, autoimmunity is not only a clinical challenge but also a conceptual gateway to reimagining preventive medicine. Interpreting autoimmune manifestations as early-system sentinel events could enable earlier recognition of resilience decline, support more targeted therapeutic intervention, and ultimately improve functional longevity.

### 4.5 Limitations and scope

Although framing autoimmunity as a sentinel of resilience collapse is conceptually integrative, it may oversimplify the diverse etiologies and age distributions of autoimmune diseases. Environmental exposures, hormonal influences, and genetic predispositions contribute substantially and may intersect with, but not be wholly explained by, resilience biology. Future research should therefore delineate disease- and age-specific resilience signatures to avoid overgeneralization of this model.

## 5 Conclusions

The evidence synthesized in this perspective highlights that the processes driving biological aging and those underlying autoimmune pathogenesis are not independent trajectories but rather intersecting outcomes of a progressive dysfunction of resilience. Biological resilience encompasses the coordinated capacity of molecular, cellular, and systemic mechanisms to preserve homeostasis in the face of stress and perturbation. When these mechanisms remain intact, transient insults can be repaired, tolerance is reinforced, and immune activation is effectively constrained. However, with advancing age, the cumulative deterioration of DNA repair, proteostasis, metabolic flexibility, and epigenetic fidelity undermines the organism’s ability to reset equilibrium after disruption. The immune system, being one of the most dynamically regulated and stress-sensitive networks, is particularly vulnerable to this decline. It is within this vulnerable context that autoimmunity emerges as a clinical manifestation of resilience collapse. While this framework provides a unifying view of aging and autoimmunity, it is important to recognize that the underlying mechanisms are heterogeneous. Not all autoimmune diseases are primarily age-driven; several arise during developmental or reproductive phases when distinct resilience mechanisms are at play. Thus, our model applies most directly to autoimmune conditions that correlate with mid-to-late-life resilience decline, rather than those with peak incidence in youth.

At the molecular level, chronic low-grade inflammation, cellular senescence, mitochondrial dysfunction, and epigenetic drift each contribute to the dismantling of tolerance mechanisms. These processes are not isolated but interconnected through feedback loops that amplify damage and compromise adaptive potential. Persistent inflammatory signaling accelerates epigenetic instability; senescent cell accumulation sustains pro-inflammatory and antigenic environments; metabolic deficits impair energy-dependent repair pathways; and disrupted non-coding RNA networks deregulate transcriptional control of immune quiescence. The convergence of these mechanisms creates a biological landscape in which autoreactive lymphocytes not only arise more frequently but also persist and expand, ultimately breaching tolerance barriers that were once resilient.

At the systems level, the decline of resilience transforms the organism from one capable of containing self-reactivity into one in which autoreactive responses propagate unchecked. The buffering capacity that in youth suppresses or extinguishes transient autoreactivity diminishes with age, permitting localized immune perturbations to escalate into chronic and systemic pathology. Organ-specific vulnerabilities further reveal how resilience collapse and autoimmunity interact. The pancreas, central nervous system, and joints illustrate tissues where intrinsic regenerative limits, metabolic demands, or mechanical stress intersect with systemic resilience erosion, resulting in disproportionate susceptibility to autoimmune disease. These observations reinforce the concept that autoimmunity is not an isolated immunological accident but the outcome of systemic destabilization across multiple physiological domains. Together, these findings highlight the importance of understanding resilience as a unifying principle in age-associated autoimmunity.

From a translational perspective, reconceptualizing autoimmunity as a sentinel of resilience decline opens new avenues for prevention and therapy. Biomarkers that integrate signals from inflammation, senescence, epigenetic drift, and metabolic stress hold promise for identifying individuals at heightened risk before overt disease onset. Such multidimensional biomarker frameworks could transform clinical practice by enabling early detection of resilience erosion and stratification of populations for preventive interventions. Therapeutically, the focus must extend beyond suppressing immune activation to fortifying the resilience pathways that sustain tolerance and repair. Senolytic therapies, metabolic enhancers such as nicotinamide adenine dinucleotide repletion, modulators of non-coding RNA networks, and interventions aimed at restoring epigenetic stability exemplify strategies that could address the underlying causes of vulnerability rather than its downstream manifestations. Importantly, such approaches may be most effective if deployed proactively, prior to irreversible tissue damage, when resilience reserves remain partially intact.

Overall, this framework suggests that autoimmunity should be viewed not only as a disorder of immune self-recognition but also as an indicator of broader systemic challenges in maintaining adaptive equilibrium. This perspective compels a shift in both scientific inquiry and clinical strategy. For research, it necessitates integrative approaches such as longitudinal multiomic profiling and systems biology modeling to unravel the causal dynamics linking resilience decline with autoimmune susceptibility. For clinical translation, it demands a reorientation from reactive disease management toward proactive resilience preservation as a means of promoting healthy longevity.

By situating autoimmunity within the broader context of resilience collapse, the boundaries between aging biology and immune pathology are redefined. This unifying approach highlights the need for interdisciplinary research that bridges geroscience, immunology, and translational medicine. Ultimately, advancing our capacity to measure, preserve, and restore resilience has the potential not only to mitigate autoimmune disease but also to extend the period of life spent in health, thereby addressing one of the central challenges of modern biomedical science. This integrative understanding may support more effective strategies to improve both immune function and overall healthspan.

## Data Availability

The original contributions presented in the study are included in the article/**Supplementary Material**, further inquiries can be directed to the corresponding author/s.
