# Supplementary material for: Cross-talk between aging resilience pathways and autoimmunity onset
Source: Front Immunol. 2025 Dec 9;16:1712575. doi: 10.3389/fimmu.2025.1712575 (PMC12722448; doi:10.3389/fimmu.2025.1712575)
Supplement: Supplementary file 1 [file DataSheet1.pdf]

Supplementary Table 1. Biomarkers of Resilience Decline with Autoimmune Predictive Value

Biomarker Class	Resilience Indicator	Mechanistic Relevance	Autoimmune Predictive/Diagnostic Value	Examples
**Epigenetic Clocks**	DNA methylation drift as a measure of biological age	Reflect cumulative resilience erosion	Predicts age-related immune instability	Horvath/Pace of Aging clocks with immune parameters
**Non-coding RNAs**	miRNA/lncRNA shifts reflect transcriptional instability	Control tolerance-related genes and cytokines	Aberrant profiles in lupus, RA, MS	miR-21, miR-146a, GAS5
**SASP Factors**	IL-6, IL-8, MMPs indicate senescent burden	Reflects systemic pro-inflammatory load	Elevated in preclinical and established autoimmunity	SASP proteomic panels
**Inflammatory Mediators**	Persistent CRP, TNF-α, IL-1β indicate inflammaging	Chronic exposure erodes tolerance	Predicts autoimmune flares and activity	Cytokine signatures
**Metabolic Markers**	NAD^+ levels, mitochondrial DNA damage, autophagy markers	Reflect energy/redox homeostasis	Link metabolic failure with autoreactive persistence	NAD^+/NADH ratio, mitophagy assays
**Composite Signatures**	Integrated scores across multiple resilience axes	Capture cross-talk of resilience pathways	Stratify patients for preventive or resilience-based therapies	Multi-omic biomarker panels
