# Supplementary material for: Cross-talk between aging resilience pathways and autoimmunity onset
Source: Front Immunol. 2025 Dec 9;16:1712575. doi: 10.3389/fimmu.2025.1712575 (PMC12722448; doi:10.3389/fimmu.2025.1712575)
Supplement: Supplementary file 2 [file DataSheet2.pdf]

Supplementary Table 2. Translational Implications: From Suppression to Resilience Enhancement

Strategy	Targeted Pathway	Mechanistic Basis of Action	Autoimmune Relevance	Example Approaches
**Senolytics**	Cellular senescence	Eliminates SASP-producing cells, reduces inflammation	Decreases autoantigen exposure and inflammatory load	Dasatinib + quercetin, navitoclax
**Metabolic Enhancers**	NAD^+/Mitochondrial function	Restores energy homeostasis, activates sirtuins	Shifts T cell balance away from pro-inflammatory phenotypes	NAD^+ boosters, nicotinamide riboside
**Epigenetic Modulators**	DNA methylation/histone modifications	Stabilizes immune tolerance-related transcriptional programs	Reduces autoreactive gene expression	DNMT activators, HDAC inhibitors
**RNA-based Therapies**	Non-coding RNA dysregulation	Rebalances microRNAs and lncRNAs controlling tolerance	Restores immune quiescence at high specificity	Anti-miR-21, GAS5 mimics
**Immunometabolic Drugs**	Autophagy/mitochondrial fitness	Promotes clearance of damaged organelles, antigens	Limits antigen persistence that sustains autoreactivity	mTOR inhibitors, AMPK activators
**Preventive Interventions**	System-wide resilience before clinical disease	Strengthens tolerance networks early	Delays autoimmune onset, reduces severity	Biomarker-guided early therapy
